# Rapid Evolution of Sex Pheromone-Producing Enzyme Expression in *Drosophila*

**DOI:** 10.1371/journal.pbio.1000168

**Published:** 2009-08-04

**Authors:** Troy R. Shirangi, Héloïse D. Dufour, Thomas M. Williams, Sean B. Carroll

**Affiliations:** 1 Howard Hughes Medical Institute, University of Wisconsin, Madison, Wisconsin, United States of America; 2 Laboratory of Molecular Biology, University of Wisconsin, Madison, Wisconsin, United States of America; University of California, Berkeley, United States of America

## Abstract

A wide range of organisms use sex pheromones to communicate with each other and to identify appropriate mating partners. While the evolution of chemical communication has been suggested to cause sexual isolation and speciation, the mechanisms that govern evolutionary transitions in sex pheromone production are poorly understood. Here, we decipher the molecular mechanisms underlying the rapid evolution in the expression of a gene involved in sex pheromone production in Drosophilid flies. Long-chain cuticular hydrocarbons (e.g., dienes) are produced female-specifically, notably via the activity of the desaturase DESAT-F, and are potent pheromones for male courtship behavior in *Drosophila melanogaster*. We show that across the genus *Drosophila*, the expression of this enzyme is correlated with long-chain diene production and has undergone an extraordinary number of evolutionary transitions, including six independent gene inactivations, three losses of expression without gene loss, and two transitions in sex-specificity. Furthermore, we show that evolutionary transitions from monomorphism to dimorphism (and its reversion) in *desatF* expression involved the gain (and the inactivation) of a binding-site for the sex-determination transcription factor, DOUBLESEX. In addition, we documented a surprising example of the gain of particular *cis*-regulatory motifs of the *desatF* locus via a set of small deletions. Together, our results suggest that frequent changes in the expression of pheromone-producing enzymes underlie evolutionary transitions in chemical communication, and reflect changing regimes of sexual selection, which may have contributed to speciation among *Drosophila*.

## Abstract 2

Rapid evolution of gene expression patterns responsible for pheromone production in 24 species of *Drosophila* was mapped to simple mutations within the regulatory domain of the *desatF* gene.

## Author Summary

Mate selection is a complex process involving communication between potential partners on many levels, such as visual, aural, and olfactory cues. Many animals use chemical signals in the form of pheromones to communicate and correctly recognize individuals of the appropriate species and sex during reproduction. Evolutionary changes in the production of these chemicals have been suggested to contribute to speciation. Yet, the molecular mechanisms governing these transitions have seldom been addressed. Here, we show that expression of the gene *desatF*, which encodes an enzyme involved in the production of the *Drosophila* pheromones known as dienes, is highly variable and rapidly evolving across *Drosophila* species. Changes in *desatF* gene expression correlate with changes in sex- and species-specific production of dienes. Further, these changes in diene production can be explained by simple modifications in the regulatory regions of the *desatF* gene, providing a molecular level understanding of the evolution of pheromone production in *Drosophila*.

## Introduction

Chemical communication is widespread in the animal world [1]. Pheromones can mediate aggregation, signal danger, attract mates, and elicit a variety of other behaviors. The species-specificity of pheromonal signals is of decisive importance in kin recognition and in sexual reproduction [2]. Evolutionary transitions in sexual communication have been suggested to govern the early stages of speciation [3]. Moreover, reproductively relevant traits such as sex pheromones are thought to evolve rapidly under sexual selection [4]. These observations raise the possibility that the genes that control how individuals communicate with each other are evolutionarily labile and may display a large degree of functional divergence across taxa.

However, the molecular mechanisms that govern how pheromone signals evolve are not understood. In principle, pheromone signaling could evolve via changes in pheromone production, chemical structure, or reception. In order to decipher how pheromone signaling may evolve, the genes that contribute to pheromone signaling and the mutations that alter it must be identified.

*Drosophila* male courtship behavior is triggered in part by female pheromones that act either by direct contact and/or by transmission over short distances. Sex pheromones in *Drosophila* are largely fatty-acid derived hydrocarbons that are present on the fly's cuticle [5]. Among Dipteran fly species, females exhibit considerable divergence in the number or position of double bonds in cuticular hydrocarbons [6]. In the *Sophophora* subgenus, females of some species, such as *D. melanogaster* [6]–[10], *D. sechellia* [6],[9],[11], and *D. erecta* [6] specifically produce long-chain dienes, which are hydrocarbons that contain two double bonds [6]. Alternatively, other species, such as *D. serrata*, *D. pseudoobscura*, and *D. persimilis* [12]–[14] produce these compounds in both sexes. Moreover, other species such as *D. simulans*, *D. mauritiana*, *D. yakuba*, *D. teissieri*, *D. orena*, and *D. santomea* [6],[7],[9],[15],[16] do not produce dienes. Cuticular hydrocarbons are suggested to have multiple roles (e.g., protection against dessication and cold resistance [17],[18]), and evolutionary transitions in the production of these compounds have been linked to reproductive isolation [9],[19]. Males from species that produce dienes dimorphically preferentially court heterospecific females that carry dienes over heterospecific females that do not harbor these compounds [9],[20].

In *D. melanogaster*, the synthesis of dienes depends in part on the product of the *desatF* gene (also known as *Fad2*) [21], a desaturase that catalyzes the addition of a second double bond in cuticular hydrocarbons. This gene is transcribed female specifically in adults [21]. The loss of desatF activity causes both a decrease in the amount of dienes, and a decrease in the attractiveness of females to males during courtship [21], suggesting a crucial role in mate recognition. Others have hypothesized that differences at the *desatF* locus may contribute to the difference in diene production between *D. simulans* and *D. sechellia* [22] and other *Drosophila* species [23].

Here, we investigate the evolution and regulation of the *desatF* gene across the subgenus *Sophophora*. First, we show that the *desatF* locus and its expression are extremely rapidly evolving across the subgenus *Drosophila*. Second, we demonstrate that the female-specific isoform of the protein encoded by the sex differentiation gene, *doublesex* (*dsx*), directly activates *desatF* expression in species that express *desatF* female-specifically. Third, we reveal that one species evolved monomorphic expression of *desatF* by functional inactivation of an ancestral DSX-binding site in the *desatF* regulatory region. And finally, we uncover an apparent case of recent stabilizing selection on *desatF* expression and show that in *D. melanogaster*, new *cis*-regulatory inputs in the *desatF* enhancer have evolved by a series of small deletions. We suggest that rapid evolution in the expression *desatF* underlies changes in the synthesis of cuticular hydrocarbons, which are likely to alter chemical communication between and within *Drosophila* species.

## Results

### *desatF* Is Expressed Female Specifically in Oenocytes

**Figure 1:**
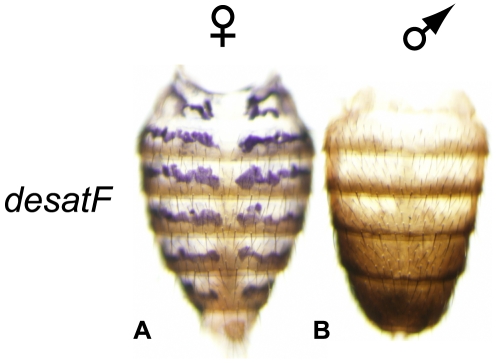
*desatF* is expressed female-specifically in *D. melanogaster* oenocytes. In situ hybridization for *desatF* performed on 4-d-old adult *D. melanogaster* flies revealed *desatF* expression in abdominal oenocytes in females (A, purple stripes) but not in males (B), These cells are the sites of cuticular hydrocarbon production.

It has been suggested that the biosynthesis of cuticular hydrocarbons takes place in specialized cells called oenocytes [21],[24],[25], which are present underneath the dorsal and ventral abdominal cuticle. Histological, confocal, and electron microscopy studies have characterized adult oenocyte cells as being organized in metameric, transverse ribbon-like stripes, that do not cross the midline and are positioned just anterior to the intersegmental region of each segment in the dorsal abdomen [26]. Adult oenocytes cells are also present in each segment of the ventral abdomen [26]. While *desatF* is known to be transcribed female-specifically, spatially restricted expression in oenocytes has not been demonstrated since all other analyses relied on reverse transcription (RT)-PCR on whole flies [21],[23]. We therefore developed an in situ hybridization protocol to visualize mRNA transcripts in adult abdomens. Consistent with previous studies, *desatF* expression is female-specific in *D. melanogaster*. Our in situ analysis revealed *desatF* expression in a pattern that is entirely consistent with previous histological descriptions [26] of adult oenocyte cells (Figure 1A, purple stripes). Moreover, desatF expression in the adult abdomen is identical to the pattern revealed by a previously characterized GAL4 driver that is active in oenocyte tissue (see “*desatF* Expression in Female Oenocytes Is Directly Activated by the Female-Specific DOUBLESEX Isoform,” below; Figure 4A and 4B, [21]). *desatF* expression in oenocyte cells is consistent with its role in diene biosynthesis. Our in situ analysis was limited to oenocyte cells and we cannot rule out expression of *desatF* in other tissues.

### Dynamic Evolutionary Changes in *desatF* Expression Correlate with Diene Production in the *Sophophora* Subgenus

**Figure 2:**
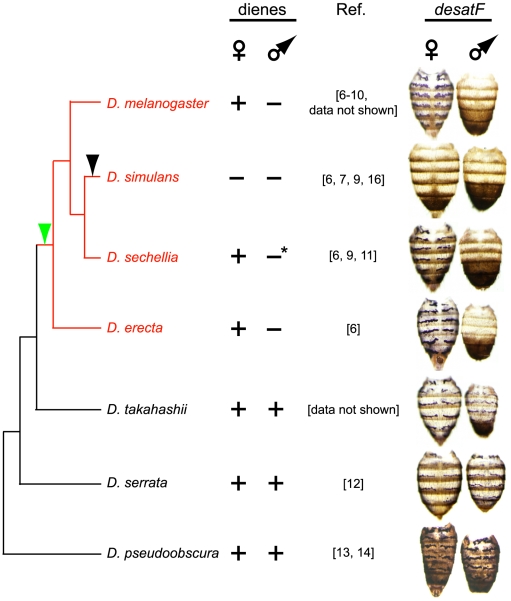
*desatF* expression correlates with evolutionary transitions in diene production from sexual monomorphism to dimorphism. Left column: phylogenetic relationships of the species used in this study. The *D. melanogaster* species subgroup is highlighted in red. Transitions in diene production from sexual monomorphism to dimorphism, and from dimorphism to amorphism, are indicated by a green and a black arrowhead, respectively. Middle column: diene production in males and females of these species. Diene production across the *Sophophora* subgenus shows several transitions: from monomorphism to dimorphism in the most recent common ancestor of the *D. melanogaster* species subgroup, and from dimorphism to amorphism in *D. simulans* (and other species not shown in the *D. melanogaster* species subgroup including *D. mauritiana*, *D. yakuba*, *D. santomea*, *D. teissieri*, and *D. orena*; summarized in Figure 7). We refer to dienes as any long-chain hydrocarbon (longer than 20 carbons) with two double bonds. Published reports of hydrocarbon profiles in these species are indicated in the column titled Ref. We have also independently validated the hydrocarbon profiles of *D. melanogaster* and *D. simulans* (unpublished data). Our analysis of *D. takahashii* males and females showed that they both produce a C23 diene (unpublished data). *, Dienes are present on *D. sechellia* males, but account for less than 2% of the total amount of cuticular hydrocarbons present on the fly [6]. The putative expression of *desatF* in *D. sechellia* males is likely to be below the detection capabilities of in situ hybridization analyses. Right column: In situ hybridization of *desatF* expression performed on 4-d-old adults. In all species studied, we observed expression of *desatF* in abdominal oenocytes (purple stripes) in accordance with their status of diene production.

The profile of cuticular diene production in the *Sophophora* subgenus exhibits several states, depending on the species (Figure 2, middle column) (unpublished data; [6]–[16]). Across this group, diene production displays an apparent transition from sexual monomorphism to dimorphism in an ancestor of the *D. melanogaster* species subgroup (Figure 2, left column, green arrowhead) and several transitions to a state of no production (Figure 2, left column, black arrow and not shown). Since *desatF* has been shown to be crucial for the production of dienes in *D. melanogaster* [21], we asked whether evolutionary changes at this locus could provide an explanation for these differences.

In order to do so, we tested whether *desatF* expression correlated with diene production. We cloned the *desatF* coding region and several kb of its upstream putative regulatory sequence from 24 species within the subgenus *Sophophora*. *desatF* expression was assessed by in situ hybridization in species where the gene lacked interruption in its reading frame. In 15 out of 24 species, *desatF* appeared to be intact (Figure 2, right column and summarized in Figure 7).

*desatF* expression correlated with diene production (Figure 2, compare the middle and right columns). In species where diene production is monomorphic (e.g., *D. pseudoobscura*, *D. persimilis*, *D. serrata*), *desatF* is expressed in oenocytes of both sexes. In species that do not produce dienes, *desatF* was either not expressed (*D. simulans*, *D. mauritiana*, *D. santomea*, and *D. teissieri*) or the gene was not intact (*D. yakuba* and *D. orena*) (Figure 2, right column and summarized in Figure 7). Finally, in species where diene production is strongly female-biased, *desatF* was expressed only in female oenocytes (*D. melanogaster*, *D. sechellia*, and *D. erecta*).

Our survey revealed that the expression of *desatF* has evolved with extraordinary rapidity (summarized in Figure 7). Of the 24 species analyzed, spanning approximately 40 million years of evolution, we uncovered ten independent evolutionary transitions (not including an additional sex-specific transition discussed below) (summarized in Figure 7). On the basis of the phylogenetic tree (adapted from [27]), *desatF* was disrupted six times by deletions and insertions of repetitive DNA (summarized in Figure 7, red bars). The expression of an intact *desatF* was lost independently at least three times (summarized in Figure 7, black bars). Based on our expression data, and in agreement with the inferences of others [23], it appears that female-specific expression was gained once at the base of the *D. melanogaster* species subgroup (Figure 2, right column).

These ten evolutionary transitions in the state of *desatF* expression among such recently diverged species mark the fastest evolving pattern of gene utilization that we know of. We were particularly interested in understanding the mechanism by which female-specific expression of *desatF* had evolved in the *D. melanogaster* species subgroup.

### The Transition from Monomorphic to a Dimorphic State of *desatF* Expression Occurred via a *cis*-Regulatory Mechanism

**Figure 3:**
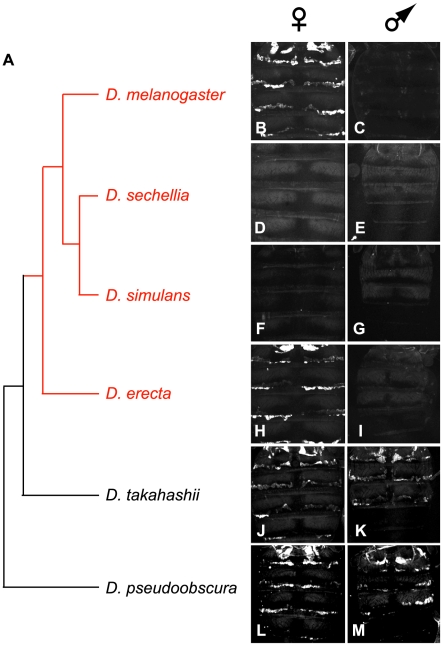
*cis*-regulatory sequence evolution governs the gain of female-specific expression of *desatF*. (A) Phylogenetic relationships of the species for which the activity of the *desatF* upstream regulatory region was assessed. Members of the *D. melanogaster* species subgroup are indicated in red. (B–M) Confocal images of the dorsal view of the abdomen from 4-d-old *D. melanogaster* females (B, D, F, H, J, and L) and males (C, E, G, I, K, and M) carrying two copies of the eGFP reporter transgene driven by the *desatF* CRE of each species indicated on the left. Note that, except for *D. sechellia*, all transgenes tested recapitulated the endogenous expression of the species they were derived from, indicating that functional differences in *cis*-regulatory sequences account for the transition from monomorphic to dimorphic expression of *desatF*.

Transitions of diene production from monomorphic to dimorphic states can be simply explained by a modification of *desatF* expression. In order to investigate the molecular basis of the regulatory changes underlying transitions in *desatF* expression, we first identified the location of a *cis*-regulatory element (CRE) within the *desatF* locus that governs gene expression in oenocytes. A screen of the 15-kb upstream intergenic region was conducted to find CREs driving enhanced green fluorescent protein (eGFP expression) in 4-d-old *D. melanogaster* oenocytes. This reporter gene assay revealed a 638-bp CRE immediately upstream of *desatF* in *D. melanogaster*, which drove eGFP expression in a manner identical to endogenous gene expression (Figures 3B and 3C, and S1, region 4).

We next tested whether differences in *desatF* expression evolved by changes in its identified CRE, or by changes in *trans*-factors that regulate its expression. We distinguished among these possibilities by analyzing the activity of orthologous CREs of *desatF* from seven species in reporter gene assays in adult *D. melanogaster*. If *cis*-regulatory sequence evolution accounts for differences in *desatF* expression, then reporter activity driven by the CREs should recapitulate the expression pattern of *desatF* in the species from which the CRE was derived. All transgenes tested (except *D. sechellia*; Figures 3D and 3E; discussed below) recapitulated endogenous temporal-, spatial-, and sex-specific-expression of *desatF* of the species from which it was derived (Figures 3F–3M). Thus, the changes underlying the transition between monomorphic and dimorphic expression of *desatF* largely occurred in the *cis*-regulatory regions of *desatF*. In order to elucidate how and when dimorphic *desatF* expression evolved, we next sought to dissect the molecular mechanisms regulating its expression in dimorphic species.

### *desatF* Expression in Female Oenocytes Is Directly Activated by the Female-Specific DOUBLESEX Isoform

**Figure 4:**
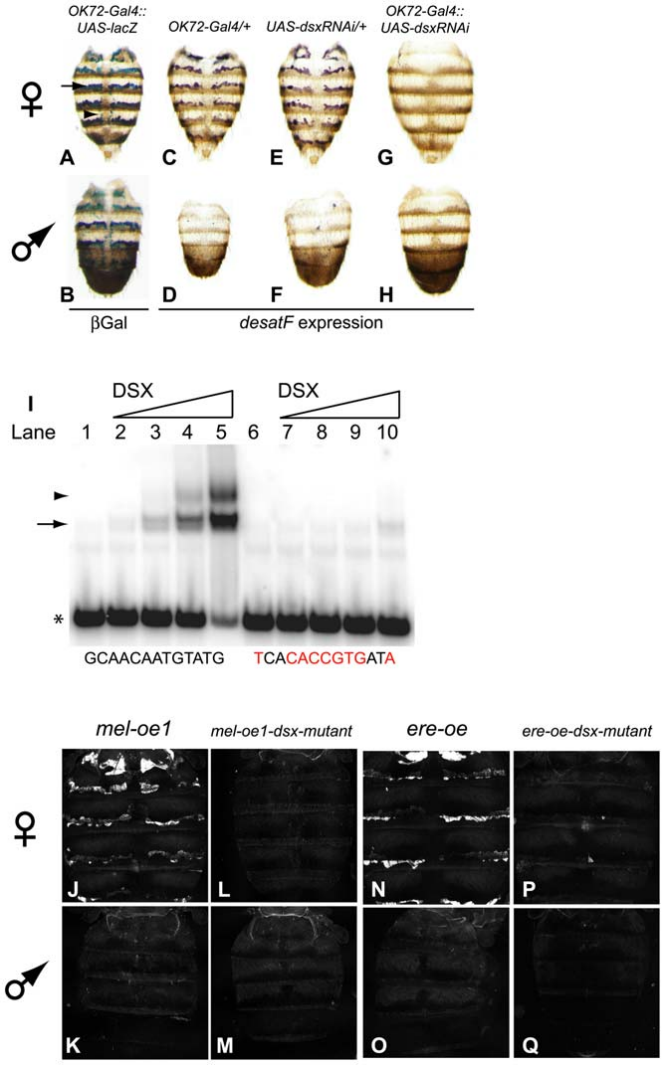
DSX-F is directly required to activate female-specific expression of *desatF* in adult oenocytes. The *OK72-Gal4* driver specifically targets oenocyte tissue in females (A) and males (B). X-Gal staining performed on 4-d-old *D. melanogaster* of the genotype indicated. Blue staining indicates that the activity of the *OK72-Gal4* driver is restricted to oenocytes (arrow) and two groups of cells (arrowhead) collinear to the dorsal vessel. These preparations retained fat body tissue, which appear to lack β-Gal activity, indicating that this driver does not target the fat body. (C–H) *In situ* hybridization for *desatF* performed on 4-d-old females (C, E, and G) and males (D, F, and H) of the *D. melanogaster* genotype indicated. Compared to the wild-type-like expression observed in control flies carrying just the GAL4 driver (C and D) and the *dsx-RNAi* (E and F) transgenes, *desatF* expression is lost in specimens expressing the *dsx-RNAi* driven by the *OK72*-GAL4 construct (G and H). (I) EMSAs were performed on annealed radiolabeled oligonucleotide probes containing the wild-type and mutant (mutated nucleotides in red) DSX-binding sites with increasing amounts of DSX-DBD protein. For probes containing the wild-type binding site, as the amount of DSX-DBD increased (lanes 1–5) a correlative increase in the amount of probe bound was observed. Protein binding was significantly reduced when the DSX-binding site was mutated (lanes 6–10). Arrow and arrowhead points to a single and pair of DSX-DBD monomers bound to the probe respectively. Asterisk identifies free probe. (J–Q) Confocal images of dorsal abdomens from 4-d-old *D. melanogaster* females (J, L, N, P) and males (K, M, O, Q) carrying two copies of an eGFP-reporter transgene. Reporter constructs are indicated at the top of the columns. The *mel-oe* (J and K) and *ere-oe* (N and O) transgenes recapitulate endogenous expression of *desatF*. However, when the DSX-binding is mutated in these constructs, they fail to drive eGFP-reporter expression in females (L and P, respectively). Reporter expression in males (M and Q, respectively) is not upregulated.

In order to elucidate factors regulating dimorphic expression of *desatF*, we first delineated the minimal *D. melanogaster* and *D. erecta* CREs capable of recapitulating sex-specific expression. In *D. melanogaster*, we identified a 271-bp element (*mel-oe2*; oe is an abbreviation for oenocyte element), upstream of *desatF*, which drives reporter activity in a pattern identical to the larger element (Figure S1). Further subdivisions led us to isolate a smaller CRE (*mel-oe1*), which also confers full reporter activity (Figures 4J and 4K). In *D. erecta*, a 710-bp CRE upstream of *desatF* (*ere-oe*) was found to recapitulate endogenous expression in transgenic reporter assays in *D. melanogaster* adults (Figures 4N, 4O, and S2).

To identify putative transcription factor binding sites within the *desatF* enhancer, we compared the sequence of *mel-oe1* with *ere-oe*. This comparison revealed a single orthologous putative binding site in both elements for the sex-specific transcription factors encoded by the *doublesex* gene (*dsx*) [28]. Female- and male-specific isoforms of the DSX transcription factor specify sexual development of soma [29]. The Yolk protein coding genes (*yp1* and *yp2*) and the *bric-a-brac* locus (*bab*) are the only known direct targets of DSX regulation [30]–[32]. In both cases, DSX proteins regulate target genes sex-specifically by modulating the function of tissue-specific activators; DSX-F enhances target gene expression in females, and DSX-M represses expression in males.

The presence of a putative DSX-binding site in the *desatF* CREs from *D. melanogaster* and *D. erecta* raised the possibility that *desatF* is a direct target of DSX regulation. This prediction was confirmed by a variety of genetic, biochemical, and transgenic reporter experiments. If DSX directly regulates *desatF*, then flies lacking DSX function should be altered in *desatF* expression. Indeed, in situ hybridization analyses indicated that females lacking DSX activity completely lacked the expression of *desatF* in adult oenocyte cells (Figure S3). Furthermore, depletion of *dsx* transcripts in oenocytes using a *UAS-dsx-RNAi* transgene driven by an oenocyte-specific Gal4 transgene (*OK72-Gal4*; Figure 4A and 4B) caused the loss of *desatF* expression in adult oenocyte cells (compare controls in Figures 4C and 4E with Figure 4G).

We next tested whether the DSX protein bound to the putative DSX binding sites in the *desatF* CREs from *D. melanogaster* and *D. erecta*. Electrophoretic mobility shift assays (EMSAs) demonstrated that the DSX-DNA-binding domain (DSX-DBD) specifically and efficiently bound the wild-type site (Figure 4I, lanes 1–5), and this binding was abolished when the DSX-binding site was mutated (Figure 4I, lanes 6–10). Furthermore, mutations in the DSX binding site of an otherwise wild-type *mel-oe1* (Figures 4J and 4K) and *ere-oe* (Figures 4N and 4O) caused a complete loss of reporter activity in vivo (Figure 4L and 4M and 4P and 4Q, respectively). Taken together, these data demonstrate that DSX-F directly activates *desatF* expression in adult oenocyte tissue. Note that while DSX-F is directly required for female-specific expression of *desatF*, it is not sufficient, and additional *cis*-regulatory inputs are also necessary for gene expression (see “*cis*-Regulatory Sites in *desatF* Were Gained by a Series of Small Deletions during *D. melanogaster* Evolution” below).

Our experiments did not indicate a repressive function for DSX-M in regulating *desatF* expression. The loss of DSX function in males did not lead to an upregulation of *desatF* in oenocyte cells (compare controls in Figure 4D and 4F with 4H), and mutations in the DSX binding site of *mel-oe1* and *ere-oe* did not lead to a gain of reporter expression in males (Figure 4M and 4Q). Thus, while DSX-F is required directly to activate *desatF* expression in females (Figure 4), unlike other known targets of DSX proteins [30]–[32], DSX-M apparently does not regulate *desatF* in males. Our studies thus demonstrate an additional mode of DSX target gene regulation.

Our phylogenetic analysis of *desatF* expression in species within the *Sophophora* subgenus led us to infer that sexually dimorphic expression arose in the ancestor of the *D. melanogaster* species subgroup (Figure 2, left column, green arrowhead). Given that DSX-F directly activates *desatF* expression in female oenocytes of *D. melanogaster* and *D. erecta*, we posited that the origin of female-specific expression arose with the DSX-binding site. In order to test this hypothesis, we investigated the ancestry of this site.

### The DSX-Binding Site at *desatF* Predates the *D. melanogaster* Species Subgroup

**Figure 5:**
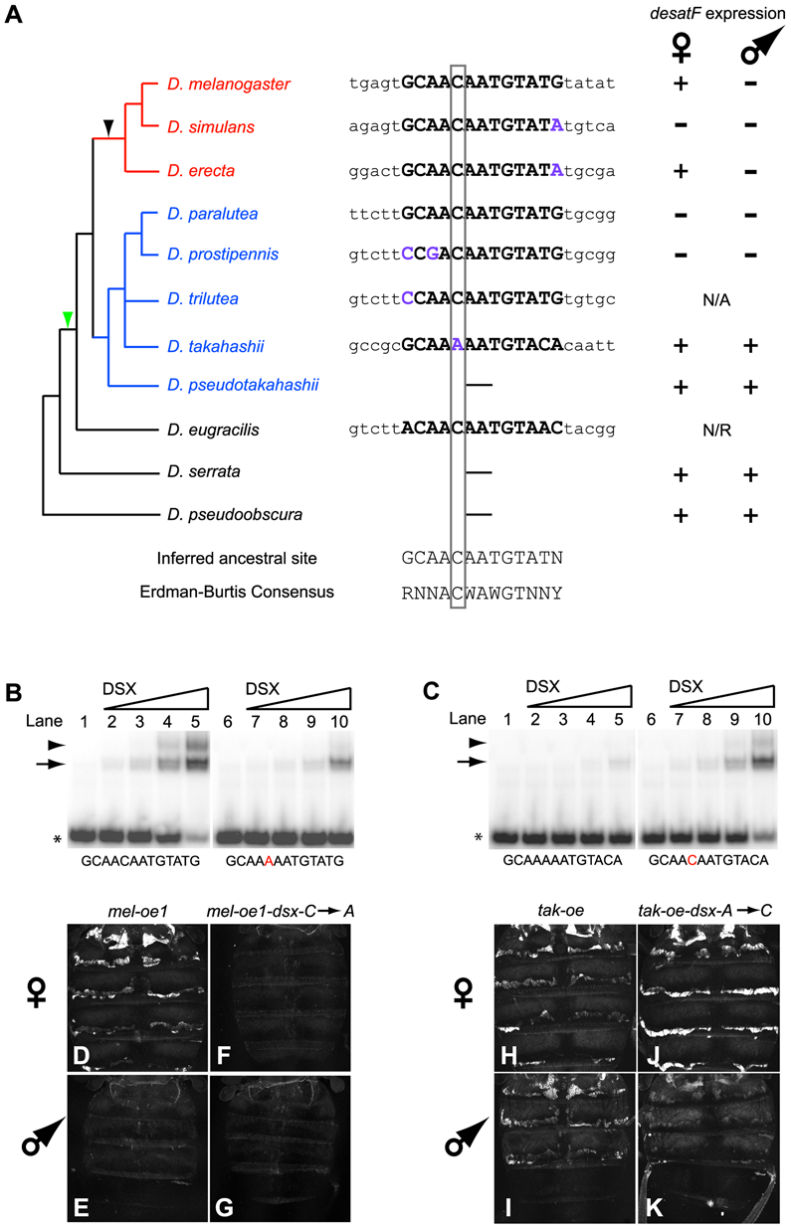
Monomorphic expression of *desatF* in *D. takahashii* evolved by functional inactivation of a DSX-binding site. (A) The DSX-binding site predates the *D. melanogaster species* subgroup (in red). Left panel: phylogenetic relationships of the species surveyed for the presence of a putative DSX-binding site (adapted from [27]). Others have positioned *D. eugracilis* and *D. ficusphila* differently in the phylogeny [63]. Our results are consistent in either case. Black arrowhead: inferred origin of *desatF* dimorphic expression, based on the phylogenetic distribution of *desatF* expression (see Figure 2). Green arrowhead: inferred origin of the DSX-binding site. Middle panel: sequences of the putative orthologous DSX-binding site of each species. Purple residues indicate positions that have diverged from the *D. melanogaster* site. Gray box identifies the critical residue within the core that has diverged in *D. takahashii*. Right panel: *desatF* expression summary. For *D. trilutea*, adult flies were not available and therefore *desatF* expression could not be assessed. *desatF* has been deleted in *D. eugracilis* and therefore assessing its expression was irrelevant. (B) EMSAs comparing the ability of the DSX-DBD protein to bind annealed radiolabeled oligonucleotide probes containing the *D. melanogaster* DSX-binding site (lanes 1–5) and a mutated version of this site (lanes 6–10) containing a C to A point mutation (in red), as found in *D. takahashii*. This mutation greatly reduced binding of the DSX-DBD. (C) EMSAs comparing the ability of the DSX-DBD protein to bind annealed radiolabeled oligonucleotide probes containing the *D. takahashii* putative DSX-binding site and a mutated version of this site containing a C in the core of the putative site instead of an A, which is found in the consensus and in the *D. melanogaster* site. In contrast to the wild-type *D. takahashii* site (lanes 1–5), where no significant binding is observed, the DSX-DBD protein binds the mutant site relatively efficiently (lanes 6–10). The arrow and arrowhead point to a single and pair of DSX-DBD monomers bound to the probe. The asterisk marks the position of the free probe. (D–K) eGFP reporter expression in abdomens of 4-d-old *D. melanogaster* females (D, F, H, and J) and males (E, G, I, and K) and carrying two copies of the transgenes indicated at the top of the columns. Introducing a C to A point mutation in the DSX-binding site of *mel-oe1* abolishes eGFP reporter expression in females (compare D and F), while leaving the absence of expression in males unchanged (compare E and G). Introducing an A to C point mutation in the putative DSX-binding site of *tak-oe* produces sexually dimorphic eGFP expression (J, K), whereas a wild-type *tak-oe* drives monomorphic expression (H, I).

If the DSX-binding site at *desatF* evolved concomitantly with the origin of dimorphic expression, then all outgroup species to the *D. melanogaster* species subgroup analyzed in our study, which display either monomorphic expression, or no expression of *desatF*, should lack an orthologous DSX-binding site in the oenocyte CRE of *desatF*. While we did not find an orthologous DSX-binding site in the outgroup species *D. pseudoobscura*, *D. persimilis*, or *D serrata*, which display monomorphic expression of *desatF*, we were surprised to find several outgroup species that contained an orthologous sequence similar to the DSX-binding site consensus [28] within the upstream regulatory region of *desatF* (Figure 5A). In *D. prostipennis*, *D. paralutea*, and *D. eugracilis*, all of which lack *desatF* expression in adult oenocytes, and in *D. takahashii*, which expresses *desatF* in both sexes, there is an orthologous sequence that matches the *D. melanogaster* DSX-binding site for at least ten out of 13 base pairs (Figure 5A, right panel). In *D. paralutea*, the orthologous site matches the *D. melanogaster* site perfectly. These data indicate that the origin of the DSX-binding site most likely predated the origin of the *D. melanogaster* species subgroup (Figure 5A, red). If true, then the origin of the DSX-binding site would be deeper in the phylogeny (Figure 5A, green arrowhead) than the origin of dimorphic expression originally inferred from our phylogenetic expression analyses (Figure 5A, black arrowhead), and those from others [23].

### Monomorphic Expression of *desatF* in *D. takahashii* Evolved by Functional Inactivation of the DSX-Binding Site

One interpretation of the observations above is that dimorphic expression of *desatF* actually arose with the acquisition of the DSX-binding site in the *desatF* CRE and was subsequently lost in the *D. takahashii* subgroup and in *D. eugracilis*. If this were true, then the state of *desatF* expression in *D. takahashii*, *D. prostipennis*, *D. paralutea*, and *D. eugracilis* evolved from an ancestor that expressed *desatF* female-specifically by direct DSX regulation. Moreover, monomorphic expression of *desatF* in *D. takahashii* would then be predicted to have evolved at least in part by loss of direct DSX regulation.

In order to test this possibility, we examined the *D. takahashii desatF* CRE and putative DSX-binding site in greater detail. We observed that the putative DSX-binding site present in the *desatF* CRE of *D. takahashii* diverges from the consensus sequence in two positions, one of which is located in the core of the site, where an A is found instead of the consensus C (Figure 5A). We note that this difference is likely to be derived in *D. takahashii* as all other species with the orthologous site contain a consensus C at this position (Figure 5A). Given that this change is located in the core of the sequence, it suggested to us that DSX proteins might not bind the *D. takahashii* site. EMSAs revealed that, in fact, the DSX-DBD failed to bind the orthologous site in *D. takahashii desatF* (Figure 5C, lanes 1–5). Furthermore, introducing the identical C-to-A mutation in the core of the *D. melanogaster* DSX-binding site greatly diminished binding of the DSX-DBD relative to wild type (Figure 5B, compare lanes 1–5 with lanes 6–10), and when introduced in an otherwise wild-type *mel-oe1* (Figures 5D and 5E), this C-to-A mutation caused a complete loss of reporter activity (Figure 5F). These results show that the putative *D. takahashii* DSX-binding site is nonfunctional.

Our data are consistent with an evolutionary scenario in which monomorphic expression of *desatF* in *D. takahashii* evolved from a dimorphic ancestor by a loss-of-function mutation in the ancestral DSX-binding site. We tested this scenario by assessing whether the restoration of a functional DSX-site by an A-to-C transition in the *D. takahashii* oenocyte CRE would result in dimorphic reporter expression. A 296-bp CRE upstream of *desatF* from *D. takahashii* is fully sufficient to recapitulate monomorphic expression of *desatF* in transgenic reporter assays in *D. melanogaster* (Figure 5H and 5I). EMSAs confirmed that an A-to-C mutation in the core of the *D. takahashii* DSX-like binding site, which converts it to the consensus sequence, is sufficient to restore binding by the DSX-DBD (Figure 5C, compare lanes 1–5 with lanes 6–10). Remarkably, this change was also sufficient to increase reporter expression in females and to decrease reporter expression in males relative to wild-type constructs (Figure 5J and 5K). This modified CRE is thus functionally dimorphic in contrast to the wild-type functionally monomorphic construct (compare Figure 5H and 5I with 5J and 5K). These results are consistent with the monomorphic expression of *desatF* in *D. takahashii* having evolved, at least in part, by the inactivation of the DSX-binding site that was present in an ancestor that expressed *desatF* female-specifically. Furthermore, this brings us to a total of two transitions in the sex-specificity of *desatF* expression (summarized in Figure 7, pink bars): a gain of dimorphism and a subsequent transition to monomorphism.

### Female-Specific Expression of *desatF* Is Consistent with Stabilizing Selection

In the course of our studies of *desatF* regulation, we were surprised to discover that while *D. melanogaster*, *D. sechellia*, and *D. erecta* express *desatF* similarly in female oenocytes (Figure 2, right column), their respective oenocyte CREs were significantly different in structure. We found that sequences orthologous to the *mel-oe2* CRE from *D. erecta* (i.e., *ere-oe2*; Figure S2) and *D. sechellia* (i.e., *sec-oe2*; Figure 3, compare 3B with 3D) failed to drive expression in transgenic reporter assays in *D. melanogaster*. These results suggested that female-specific *desatF* expression in *D. erecta* and *D. sechellia* rely at least in part on different *cis*-regulatory sites than those characterized in *D. melanogaster*.

Indeed, for *D. erecta*, additional sequences outside the *ere-oe2* region are required for reporter activity. By extending the 5′-end of *ere-oe2* by 190 bp (*ere-oe3*; Figure S2), we obtained full reporter activity in *D. melanogaster* female oenocytes. Importantly, this 190-bp region, by itself, is not sufficient for reporter function. Furthermore, the orthologous region from *D. melanogaster* is clearly not required for CRE function, as *mel-oe2* is a fully functional CRE despite lacking the orthologous 190-bp region (Figure S1). Thus, the functional *D. melanogaster* and *D. erecta* CREs share common necessary features (e.g., the DSX-binding site), but also exhibit critical differences (e.g., the 190-bp region required for *D. erecta* CRE activity).

For *D. sechellia*, an exhaustive search of all intergenic sequences upstream and downstream of *desatF* failed to identify a region that drove reporter expression in the *D. melanogaster* genetic background (unpublished data). This may indicate that the *D. sechellia* CRE for oenocyte expression is located outside of the regions searched and/or that there are *trans*-acting regulatory differences between the species, which are key for *desatF* expression in *D. sechellia* females. We note there is a putative DSX-binding site in the *D. sechellia desatF* CRE that matches the consensus site described for *D. melanogaster* [28]. Thus the absence of reporter expression from the *D. sechellia desatF* CRE in the *D. melanogaster* molecular background is not likely to be due to a failure of DSX to bind the site. Together, our data indicate that *D. melanogaster*, *D. sechellia*, and *D. erecta* express *desatF* female-specifically in part by distinct *cis*-regulatory mechanisms.

One explanation for these results is that stabilizing selection has maintained phenotypic constancy for *desatF* expression while mutational turnover of functionally important sites has taken place. This phenomenon has been previously reported [33]–[38], but described for species that have diverged over relatively long periods of evolutionary time (40 millions y or more). It is surprising that drastic alterations in the *cis*-regulatory mechanisms at *desatF* occurred in a short period of time (*D. melanogaster* and *D. sechellia* diverged only 2–3 million y ago). More detailed investigation of the *D. melanogaster* CRE uncovered *cis*-regulatory sites specific to this species and important for *desatF* female-specific expression.

### *cis*-Regulatory Sites in *desatF* Were Gained by a Series of Small Deletions during *D. melanogaster* Evolution

**Figure 6:**
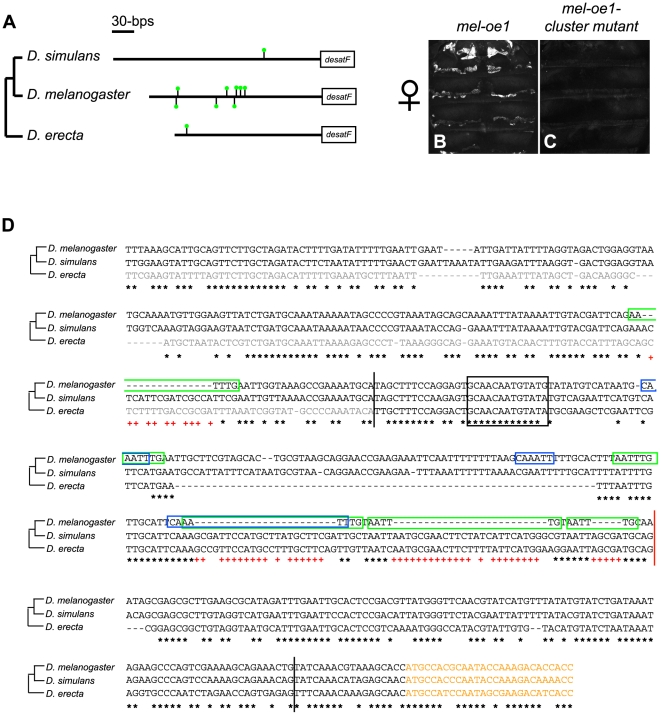
*Cis*-regulatory information was gained by deletion during *D. melanogaster* evolution. (A) The AATTTG motif is statistically overrepresented in *mel*-oe2. Schematic representation of the AATTTG motifs (green dot over black bar) in *mel-oe2* and its orthologous sequence from *D.simulans* and *D. erecta*. (B and C) eGFP reporter expression in abdomens of 4-d-old *D. melanogaster* females carrying two copies of the transgenes indicated at the top of the images. The introduction of point-mutations in the clustered AATTTG motifs of *mel-oe1* (C) abolishes eGFP reporter expression driven in female oenocytes by a wild-type *mel-oe1* (B).The absence of reporter activity in males is not altered by these mutations (not shown). (D) Alignment of the desatF upstream region from *D. melanogaster*, *D. simulans*, and *D. erecta*. *mel-oe2* and its orthologous sequences from *D. simulans* and *D. erecta* are delineated by vertical black bars. The vertical red bar indicates the 3′ end of *mel-oe1*. *mel-oe1* and *mel-oe2* begin at the same 5′ position. AATTTG motifs are boxed in green (forward orientation) and blue (reverse orientation). Black stars (*) indicate conservation among the three species. The red plus sign (+) indicates conservation between *D. simulans* and *D. erecta*. The beginning of the coding region is in yellow. The *D. erecta* 190-bp sequence that is necessary (in addition to *ere-oe2*) to produce a construct capable of full reporter activity in *D. melanogaster* female oenocytes is represented in grey. Note the very well conserved indels in *D. simulans* and *D. erecta*, which disrupt each of the three AATTTG motifs in the cluster, indicating that those hexamer motifs evolved by a series of small deletions.

While investigating the *cis*-regulatory differences at *desatF* between the three dimorphic species, we uncovered an unusual feature specific to *D. melanogaster*. We found eight copies of the hexamer AATTTG in its upstream regulatory region (Figure 6A), i.e., within *mel-oe1* and -*oe2*, which was overrepresented with high statistical significance (occurrence *p* = 0.0000051; from the Oligo-analysis program [39]). The motif does not match any binding site consensus to our knowledge. In order to test whether these motifs were functionally relevant, we introduced point mutations in six of these motifs in an otherwise wild-type *mel-oe2* CRE. This led to a complete loss of reporter activity in female oenocytes (unpublished data), indicating that these motifs are indeed necessary for CRE function.

Most of these hexamer motifs were absent in the orthologous region from *D. erecta*, *D. sechellia* (Figure 6A), and all other species examined in our study (unpublished data), suggesting new *cis*-regulatory sites evolved recently in the *D. melanogaster* lineage. The evolution of these hexamer motifs could have occurred through a variety of mechanisms [40]. Rearrangement events such as transposition and duplication, and binding site formation by point mutation, are the two main modes by which new *cis*-regulatory content has been suggested to evolve [41]–[43]. In order to understand the mutational path that produced these hexamer motifs at *desatF* in *D. melanogaster*, we compared the *mel-oe2* sequence to its ortholog in closely related species, *D. simulans* and *D. erecta*. Of particular interest is the cluster of three motifs in the forward direction in *mel-oe2* (Figure 6A). A sequence alignment of *mel-oe2* and its ortholog from *D. erecta* and *D. simulans* revealed that, except for the hexamers, this region is largely conserved, excluding a transposition event (Figure 6D; see below). Closer scrutiny of the alignment in the hexamer region revealed in *D. simulans* and *D. erecta* the presence of common insertions/deletions disrupting each of the three hexamer motifs in the cluster (Figure 6D). On the basis of the phylogenetic relationships among these three species, we infer that the three hexamer motifs were gained during *D. melanogaster* evolution by a series of nonidentical small deletions.

We tested if these three particular motifs were required for CRE function by introducing point mutations in the *mel-oe1* CRE. We found that they caused a complete loss of reporter activity (Figure 6B and 6C). To test whether the AT content of these motifs, instead of their sequence, could explain their functional relevance, we mutated the three sites in *mel-oe1* without altering their AT percentage. This construct also failed to produce reporter activity (Figure S4). We suggest that these hexamer sequences are binding sites for a transcription factor and that they evolved via a series of small deletions.

## Discussion

Pheromone differences between closely related species of Lepidoptera and Diptera suggest that their production is rapidly evolving [6],[44],[45]. While rapidly evolving traits have been characterized many times, especially regarding sexually related traits [46],[47], few studies, to our knowledge, have identified the genes and mutations that give rise to these evolutionary changes [32]. We have investigated the evolution of the mechanisms that govern the production of pheromonal signals between males and females during *Drosophila* courtship. Our studies provide several insights into the molecular mechanisms of pheromone signal evolution.

**Figure 7:**
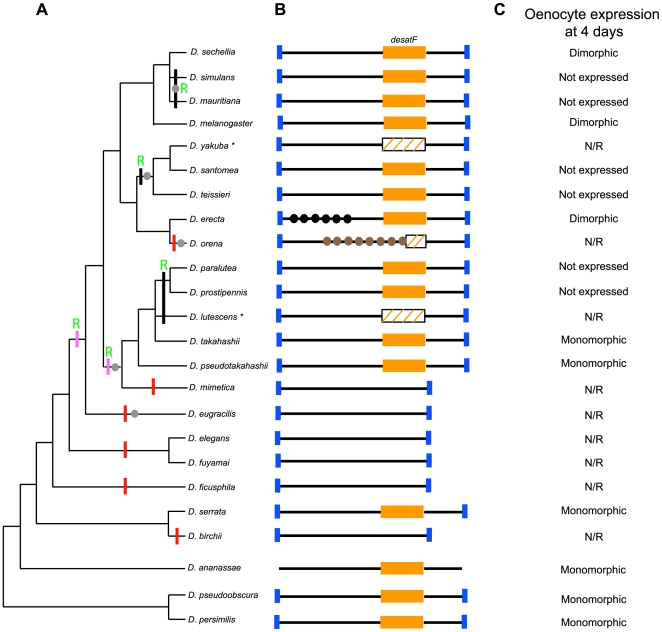
The *desatF* locus is rapidly evolving. (A) Phylogenetic relationships of the 24 species surveyed (adapted from [27]). (B) Schematic of the *desatF* locus in these species. The blue rectangles indicate the landmarks used in cloning. In *D. ananassae*, *desatF* was found in the genome, but not in synteny, which is indicated by the absence of the blue rectangles. The orange boxes indicate the coding region. A striped box indicates a mutation in the coding region leading to a loss of function of the protein (frameshift or nonsense mutation). Black and brown full circles represent regions with repetitive DNA. The six independent gene losses are indicated by red bars. Regulatory losses of expression without gene inactivation are marked by a black bar. Modifications in the sex-specificity of *desatF* expression are represented by a pink bar. Green “R” refers to regulatory transitions. Altogether, 11 independent evolutionary changes in *desatF* expression occurred in the approximate 40 millions y during which these species evolved. *, Note that the gene inactivations in *D. yakuba* and *D. lutescens* are not counted as such in our tally. In *D. yakuba*, the regulatory loss of *desatF* expression appears to have preceded the pseudogenization event. In *D. lutescens*, the ambiguous phylogenetic relationships in the clade prevents the accurate inference of transitions. Grey full circles indicate independent losses of dimorphism. (C) Status of *desatF* expression in oenocytes in 4-d-old adults aged. N/R: not relevant (because the gene was not functional based on sequence information).

We have shown that the *desatF* gene is rapidly evolving in the subgenus *Sophophora*. We found that changes in the *desatF* expression have evolved numerous independent times within 40 million y of *Drosophila* evolution, including six independent instances of gene loss, two modifications in sex-specific expression, and three independent losses of expression without gene loss. Altogether, among 24 species surveyed, we observed 11 transitions in the state of *desatF* expression (summarized in Figure 7). *desatF* has also been duplicated in some *Drosophila* species [23]. Our results reveal that the evolution of the *desatF* gene is extraordinarily dynamic, and displays, to our knowledge, the fastest evolving pattern of gene utilization observed to date.

We identified the CRE regulating *desatF* expression in *D. melanogaster* oenocytes and characterized DSX-F as a necessary and direct input for its female-specific expression. We found that transitions from sexual monomorphism to dimorphism, and the reverse, rely at least in part on the gain and loss of direct DSX regulation. Remarkably, simple evolutionary changes in *cis*-regulatory sequences were sufficient to explain the transition of *desatF* expression from dimorphism to monomorphism, as seen in *D. takahashii*.

Because *desatF* and dienes contribute strongly to *D. melanogaster* mating behaviors, it is likely that dimorphic expression of *desatF* is under sexual selection. Since the gain of female-specific expression, we count five losses of sexual dimorphism, which includes one transition to monomorphism and four transitions to amorphism (Figure 7, black dots). What does this pattern of evolutionary change suggest? The loss of sexually selected traits is widespread [48]. This pattern of frequent trait loss may be an indication of relaxed selection resulting from rapidly shifting regimes of sexual selection. For example, since fly courtship is regulated by multiple sensory cues (visual, chemosensory, auditory, etc), the modality that is under sexual selection may change, leading to trait loss.

### Regulatory Evolution as a Mechanism to Evolve Sex-Specificity

A long-standing question in evolutionary biology is how sexually dimorphic traits evolve [49]. For example, monomorphic patterns can evolve from dimorphic patterns and vice versa, however, the molecular mechanisms that govern these transitions have seldom been addressed. In Lepidoptera and Diptera, duplication or structural changes of genes encoding desaturases have been suggested to [23] or shown to contribute to evolutionary alterations in pheromone signals [50]–[52], however, none of these phenomena alone could account for evolutionary transitions in sex-specificity of pheromone production. Here, we have provided evidence that *cis*-regulatory sequence evolution led to transitions from monomorphic to dimorphic expression of *desatF*, and its reversion, and concomitant changes in diene production.

By pinpointing one of these transitions at the level of individual base-pairs, we propose that monomorphic expression of *desatF* in *D. takahashii* evolved from a dimorphic ancestor through a derived mutation in a single critical residue inactivating the orthologous DSX-binding site (Figure 4). A simple model for the origin of monomorphic gene expression is that a mutation in the DSX-binding site abrogated repression by DSX-M, in turn, up-regulating *desatF* expression in males. Furthermore, the loss of regulation by DSX-F would lead to a decrease in *desatF* expression in females. These alterations, together, would produce monomorphic expression of *desatF* in *D. takahashii*. However, we note that this model is at odds with our finding that DSX-M appears to not regulate *desatF* in *D. melanogaster*. This suggests that the ancestor of *D. melanogaster* and *D. takahashii* regulated *desatF* dimorphically by either a *D. melanogaster*-like mechanism, or by a mode that involved repression by DSX-M. While we currently cannot polarize these possibilities, both models implicate the inactivation of the ancestral DSX-binding site as a necessary step in the transition to monomorphic expression.

### *desatF* and Speciation

In order to understand the mechanisms that drive speciation, the genetic changes that lead to reproductive isolation must be elucidated. It has been suggested that, “speciation genes are those that contribute to reproductive isolation, often in the form of hybrid inviability, sterility or behavioral aberration” [53]. While progress has been made in identifying genes that contribute to postzygotic isolation (such as *Xmrk2* [54],[55], *OdsH* [53], *Nup96* [56], see review [57]), little is known of genes that contribute to prezygotic isolation. We suggest that *desatF* could be one such gene.

There is evidence that diene production contributes to reproductive isolation. For example, it has been documented that dienes inhibit *D. simulans* male courtship behavior [58]. Moreover, *D. simulans*/*D. melanogaster* hybrid females lacking *desatF* expression elicit greater levels of courtship activity from *D. simulans* males, relative to hybrids expressing *desatF* female-specifically [59]. Taken together, these data indicate that expression of *desatF* and the production of dienes in *D. melanogaster* females contribute to the reproductive isolation between these sibling species.

*desatF* expression has evolved numerous times during *Drosophila* evolution. If, as others have suggested [12], transitions in dienes contribute to sexual behavior in species other than *D. melanogaster*, then the contribution of *desatF* to speciation may be widespread.

## Materials and Methods

### Fly Stocks

Wild-type stocks were obtained from the University of California, San Diego (UCSD) stock center (see Table S1). Gal4-UAS analyses were performed using the following lines: *OK72-Gal4* was obtained from the Bloomington Stock Center; *dsx^1^ p* ^p^, *UAS-dsxRNAi*, and *UAS-lacZ* were provided by M. McKeown (Brown University).

### Imaging of Fly Abdomens

Images of in situ hybridizations and X-Gal stained adult abdomens were taken using an Olympus SZX16 Stereo Microscope equipped with an Olympus DP71 microscope digital camera. Adult transgenic eGFP-reporter line samples were imaged using an Olympus Fluoview FV 1000 confocal microscope and software. Wings and head were removed from 4-d-old adults, which were then mounted in Halocarbon 700 oil for confocal analysis.

### Sequence Analysis of Orthologous *desatF* Loci

Sequences for *D. melanogaster*, *D. simulans*, *D. sechellia*, *D. yakuba*, *D. erecta*, *D. ananassae*, and *D. pseudoobscura* were obtained from their respective genome databases. All other sequences were obtained by cloning and sequencing of orthologous sequences using genomic DNA prepared from species stocks obtained from the UCSD *Drosophila* stock center (see Table S1). Sequences were PCR amplified using different sets of degenerate primers and then fused to give rise to the sequence of the whole locus. Details are available upon request to the authors. Novel sequences have been deposited in GenBank (http://www.ncbi.nlm.nih.gov/Genbank, submission numbers are listed in Table S1). Orthologous sequences were aligned using ClustalW2 [60] with subsequent manual alignment in problematic regions. We used the GenePalette program to analyze our sequences (www.genepalette.org). We used Oligo-analysis to look for overrepresented motifs in our sequence [39]. This program calculates the probability that the analyzed sequence contains an oligonucleotide sequence at a frequency greater than that expected at random.

### In Situ Hybridization on Adult Abdomens

In situ hybridization was performed as previously described [61] with minor modifications. The complete adult abdominal in situ protocol is available at http://www.molbio.wisc.edu/carroll/. Primers used to amplify probes are listed in Table S2.

### DNA-Binding Analyses

EMSAs were performed as previously described [27],[32]. PAGE-purified oligos used in EMSAs are listed in Table S3.

### Transgenic Fly Production

All transgenic lines were produced by using the Phage ϕC31 Integrase system. Embryos from flies containing the *X*-chromosome *attP* docking site VK00046 [62] were injected as previously described [32]. Primers used to clone the constructs are listed in Table S4.

## Supporting Information

Figure S1 **Screen of the** ***D. melanogaster desatF*** **locus for CREs.** Numbers and adjacent bars indicate the *desatF* locus region surveyed in a given reporter construct. Green bars represent the regions that drove eGFP reporter activity in oenocytes in 4-d-old *D. melanogaster* flies, black bars represent regions that did not drive activity. Scale in base pairs is boxed. Additional information on the numbered eGFP-reporter gene constructs is in Table S4.
(0.78 MB EPS)

Figure S2 **Screen of the** ***D. erecta desatF*** **locus for active CREs.** Numbers and adjacent bars indicate the *desatF* locus region surveyed in a given reporter construct. Green bars represent the regions that drove eGFP reporter activity in oenocytes in 4-d-old *D. melanogaster* flies, black bars represent regions that did not show activity. Scale is boxed. Additional information on the numbered eGFP-reporter gene constructs can be found in Table S4.
(0.75 MB EPS)

Figure S3 **DSX-F is required genetically to activate female-specific expression of** ***desatF*** **in adult oenocytes.** In situ hybridization for *desatF* performed on 4-d-old *D. melanogaster*. *dsx* genotypes are indicated at the top of the columns, and sexual genotypes on the side. Compared to the heterozygous null controls (*dsx^1^/TM6B*, left panels) displaying a wild type pattern of *desatF* expression, *dsx* homozygous null mutants (*dsx^1^*, right panels) don't show female specific expression (upper right panel), nor upregulation in males (bottom right panel).
(3.76 MB EPS)

Figure S4 **The clustered AATTTG motifs contain regulatory information.** eGFP reporter expression in abdomens of 4-d-old *D. melanogaster* flies carrying two copies of the transgenes indicated at the top of the images. The introduction of point-mutations that conserve AT content in the clustered AATTTG motifs of *mel-oe1* (right panel) abolished eGFP reporter expression driven in female oenocytes by a wild-type *mel-oe1* (left panel). This result indicate that rather than being important for structural conformation of the enhancer, those hexamers are more likely binding sites for a transcription factor.
(2.44 MB TIF)

Table S1 **List of the** ***Drosophila*** **species used in this study.** The middle column refers to the UCSD stock center number. The right column refers to the GenBank accession number for the sequence orthologous to the *D. melanogaster desatF* locus. In addition, *D. mimetica* and *D. trilutea* DNA was obtained from H. Malik. The sequences were referenced respectively as FJ869331 and FJ869337.
(0.04 MB DOC)

Table S2 **Primers used to amplify probes for in situ hybridizations.** The close proximity of some species allowed cross-hybridization. The *D. melanogaster* probe was hence also used on *D. mauritiana*, *D. simulans*, and *D. sechelllia*. The *D. santomea* probe was also used on *D. teissieri*. The *D. pseudoobscura* probe was also used on *D. persimilis*.
(0.05 MB DOC)

Table S3 **Top strand EMSA oligonucleotides probes used in this study.** The putative DSX-binding site is in bold.
(0.03 MB DOC)

Table S4 **Primers used to PCR amplify CREs used in this study.** All PCR products were cloned into the S3aG EGFP reporter vector described in [32] using the restriction enzymes AscI and SbfI.
(0.06 MB DOC)

## Abbreviations

CRE: *cis*-regulatory element
DSX-DBD: DOUBLESEX DNA binding domain
DSX-F: female specific DOUBLESEX isoform
DSX-M: male specific DOUBLESEX isoform
eGFP: enhanced green fluorescent protein
EMSA: electrophoretic mobility shift assay
oe: oenocyte element
