# Supplementary figures and images for: Rapid Evolution of Sex Pheromone-Producing Enzyme Expression in Drosophila
Source: PLoS Biol. 2009 Aug 4;7(8):e1000168. doi: 10.1371/journal.pbio.1000168 (PMC2711336; doi:10.1371/journal.pbio.1000168)

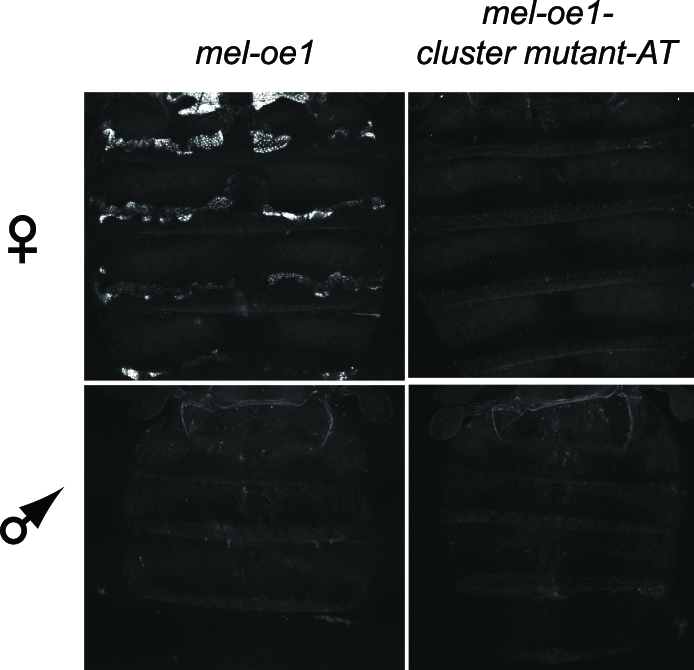

Supplement: Figure S4 — The clustered AATTTG motifs contain regulatory information. eGFP reporter expression in abdomens of 4-d-old D. melanogaster flies carrying two copies of the transgenes indicated at the top of the images. The introduction of point-mutations that conserve AT content in the clustered AATTTG motifs of mel-oe1 (right panel) abolished eGFP reporter expression driven in female oenocytes by a wild-type mel-oe1 (left panel). This result indicate that rather than being important for structural conformation of the enhancer, those hexamers are more likely binding sites for a transcription factor. (2.44 MB TIF) [file pbio.1000168.s004.tif]
