# Supplementary material for: Rapid Evolution of Sex Pheromone-Producing Enzyme Expression in Drosophila
Source: PLoS Biol. 2009 Aug 4;7(8):e1000168. doi: 10.1371/journal.pbio.1000168 (PMC2711336; doi:10.1371/journal.pbio.1000168)
Supplement: Table S1 — List of the Drosophila species used in this study. The middle column refers to the UCSD stock center number. The right column refers to the GenBank accession number for the sequence orthologous to the D. melanogaster desatF locus. In addition, D. mimetica and D. trilutea DNA was obtained from H. Malik. The sequences were referenced respectively as FJ869331 and FJ869337. (0.04 MB DOC) [file pbio.1000168.s005.doc]

| **Species** | **Origin of the species** | **Gen Bank accession number** |
| --- | --- | --- |
| *D. melanogaster* | CantonS |  |
| *D. simulans* | Florida city |  |
| *D. mauritiana* | D. maur^01^ | FJ869330 |
| *D. sechellia* | Cousin Island |  |
| *D. yakuba* | 14021-0261.01 |  |
| *D. santomea* | STO.4 | FJ869323 |
| *D. teissieri* | 14021‑0257.00 | FJ869324 |
| *D. erecta* | 14021-0224.01 |  |
| *D. orena* | 14021-0245.01 | FJ869333 |
| *D. paralutea* | 14022-0281.00 | FJ869325 |
| *D. prostipennis* | 14022-0291.00 | FJ869326 |
| *D. lutescens* | 14022-027100 | FJ869327 |
| *D. takahashii* | 14022-0311.07 | FJ869328 |
| *D. pseudotakahashii* | 14022-0301.01 | FJ869329 |
| *D. eugracilis* | 14026-0451.05 | FJ872371 |
| *D. elegans* | 14027-0461.01 | FJ869336 |
| *D. fuyamai* | 14029-0011.00 | FJ869332 |
| *D. ficusphila* | 14025-0441.01 | FJ869335 |
| *D. ananassae* | 14024-0371.13 |  |
| *D. serrata* | 14028‑0681.03 | FJ869334 |
| *D. birchii* | 14028‑0521.00 | FJ869338 |
| *D. persimilis* | 14011-0111.49 |  |
| *D. pseudoobscura* | 14011-0121.87 |  |

**Table S1. List of the Drosophila species used in this study.**

The middle column refers to the UCSD stock center number. The right column refers to the GenBank accession number for the sequence orthologous to the *D. melanogaster* *desatF* locus. In addition, D. mimetica and D. trilutea DNA was obtained from H. Malik. The sequences were referenced respectively as FJ869331 and FJ869337.
