# Supplementary material for: Rapid Evolution of Sex Pheromone-Producing Enzyme Expression in Drosophila
Source: PLoS Biol. 2009 Aug 4;7(8):e1000168. doi: 10.1371/journal.pbio.1000168 (PMC2711336; doi:10.1371/journal.pbio.1000168)
Supplement: Table S2 — Primers used to amplify probes for in situ hybridizations. The close proximity of some species allowed cross-hybridization. The D. melanogaster probe was hence also used on D. mauritiana, D. simulans, and D. sechelllia. The D. santomea probe was also used on D. teissieri. The D. pseudoobscura probe was also used on D. persimilis. (0.05 MB DOC) [file pbio.1000168.s006.doc]

| **Species** | **Forward Primer** | **Reverse Primer** |
| --- | --- | --- |
| *D. melanogaster* | 5‘‑ATGCCACGCAATACCAAAGAC-3’ | 5‘‑GCCCACARRCGATGGGCGCC-3’ |
| *D. santomea* | 5‘‑GCAACATGCCACCCAATGGCAAAGAC-3’ | 5‘‑CATCGCTTGTGGGCACATCGCAC-3’ |
| *D. erecta* | 5‘‑GCAACATGCCATCCAATAGCGAAGAC-3’ | 5‘‑GTGCGATGGGCCCACAAGCGGTG-3’ |
| *D. paralutea* | 5‘‑RWGCACSATGCCACCCAATACC-3’ | 5‘‑GTGCGATGGGCCCACAAGCGATG-3’ |
| *D. prostipennis* | 5‘‑RWGCACSATGCCACCCAATACC-3’ | 5‘‑GTGCGATGGGCCCACAAGCGATG-3’ |
| *D. takahashii* | 5‘‑RWGCACSATGCCACCCAATACC-3’ | 5‘‑GTGCGATGGGCCCACAAGCGATG-3’ |
| *D. pseudotakahashii* | 5‘‑RWGCACSATGCCACCCAATACC-3’ | 5‘‑GTGCGATGGGCCCACAACCGATG-3’ |
| *D. serrata* | 5‘‑[GTATGCCACCCAACGGCGACG](https://www.idtdna.com/OrderStatus/                                                                SpecSheet.aspx?OrderNum=4718094&MfgID=37127642&MfgLocID=1&ProdID=1213&position=8)-3’ | 5‘‑[GCGAACAGACGATGGGCGCC](https://www.idtdna.com/OrderStatus/                                                                SpecSheet.aspx?OrderNum=4718094&MfgID=37127643&MfgLocID=1&ProdID=1213&position=9)-3’ |
| *D. ananassae* | 5‘‑GACCAAGAATGCCTCCAAATAGTAACG-3’ | 5‘‑CGGTGCGACCACAGGCGATG-3’ |
| *D. pseudoobscura* | 5‘‑GCACGATGCCACCCAATAGCGATC-3’ | 5‘‑CGATGCGCCCACAGGCGATG-3’ |

**Table S2: Primers used to amplify probes for *in situ* hybridizations.**

The close proximity of some species allowed cross-hybridization. The *D. melanogaster* probe was hence also used on *D. mauritiana*, *D. simulans* and *D. sechelllia*. The *D. santomea* probe was also used on *D. teissieri*. The *D. pseudoobscura* probe was also used on *D. persimilis*.
