# Supplementary material for: Rapid Evolution of Sex Pheromone-Producing Enzyme Expression in Drosophila
Source: PLoS Biol. 2009 Aug 4;7(8):e1000168. doi: 10.1371/journal.pbio.1000168 (PMC2711336; doi:10.1371/journal.pbio.1000168)
Supplement: Table S3 — Top strand EMSA oligonucleotides probes used in this study. The putative DSX-binding site is in bold. (0.03 MB DOC) [file pbio.1000168.s007.doc]

| **Construct and Figure Number** | **EMSA Probe (Top strand)** |
| --- | --- |
| *D. melanogaster*, wild-type (Figure 4I, 5B) | 5‘‑CGAAAATGCATAGCTTTCCAGGAGT**GCAACAATGTATG**TATATGTCATAATGCAAATTTGAAT‑3’ |
| *D. melanogaster*, mutant (Figure 4I) | 5‘‑CGAAAATGCATAGCTTTCCAGGAGT**TCACACCGTGATA**TATATGTCATAATGCAAATTTGAAT‑3’ |
| *D. melanogaster*, mutant (Figure 5B) | 5‘‑CGAAAATGCATAGCTTTCCAGGAGT**GCAAAAATGTATG**TATATGTCATAATGCAAATTTGAAT‑3’ |
| *D. takahashii*, wild-type (Figure 5C) | 5‘‑TCTACGTAAACAAATTTTCAGCCGC**GCAAAAATGTACA**CAATTGCGGAAGTTTATCAGGAATT‑3’ |
| *D. takahashii,* mutant (Figure 5C) | 5‘‑TCTACGTAAACAAATTTTCAGCCGC**GCAACAATGTACA**CAATTGCGGAAGTTTATCAGGAATT‑3’ |

**Table S3. Top Strand EMSA oligonucleotides probes used in in this study.** The putative DSX-binding site is in bold.
