# Supplementary material for: Rapid Evolution of Sex Pheromone-Producing Enzyme Expression in Drosophila
Source: PLoS Biol. 2009 Aug 4;7(8):e1000168. doi: 10.1371/journal.pbio.1000168 (PMC2711336; doi:10.1371/journal.pbio.1000168)
Supplement: Table S4 — Primers used to PCR amplify CREs used in this study. All PCR products were cloned into the S3aG EGFP reporter vector described in [32] using the restriction enzymes AscI and SbfI. (0.06 MB DOC) [file pbio.1000168.s008.doc]

| **Construct** | **Figure** | **Forward Primer** | **Reverse Primer** |
| --- | --- | --- | --- |
| 1 | S2 | 5‘‑GGCAGAGACCTGCAATCTGGC‑3’ | 5‘‑TCAAAAATCGCAGCGGCAAC‑3’ |
| 2 | S2 | 5‘‑TTGCTACTCTCGATGTTGCTG‑3’ | 5‘‑TTGCGGTGATTGGCAGCAAC‑3’ |
| 3 | S2 | 5‘‑CGCTCGATAGTTTTGGTTTG‑3’ | 5‘‑GAAAACTGAGTGGCTGTTTG‑3’ |
| 4 | 3, S2 | 5‘‑CATCTACTGTCTGCACGCAG‑3’ | 5‘‑CAGTTTCTGCTTTTCGACTGGGC‑3’ |
| 5 | S2 | 5‘‑CATCTACTGTCTGCACGCAG‑3’ | 5‘‑GGAAAGCTATGCATTTTCGG‑3’ |
| 6 | S2 | 5‘‑GAGGTAATGCAAAATGTTGG‑3’ | 5‘‑CTTACGCAGTGCTACGAATG‑3’ |
| 7 (*mel-oe2*) | S2 | 5‘‑TAGCTTTCCCTGAGTGCAAC‑3’ | 5‘‑CAGTTTCTGCTTTTCGACTGGGC‑3’ |
| 8 (*mel-oe1*) | 4, 5, 6, S2, S5 | 5‘‑TAGCTTTCCCTGAGTGCAAC‑3’ | 5‘‑TCGCTATTTGCAAATTACAAATTAC‑3’ |
| 9 | S3 | 5‘‑GAATTCCAGCATTTTCACCTG‑3’ | 5‘‑GAATTCGAGCTTCGCATATAC‑3’ |
| 10 (*ere-oe*) | 3, 4, S3 | 5‘‑GAGGTATGCCACTTTTAAG‑3’ | 5‘‑GCTCTTTGTTTGAAACTCTCACTGG‑3’ |
| 11 (*ere-oe3*) | S3 | 5‘‑CATTCGAAGTATTTTAGTTC‑3’ | 5‘‑GCTCTTTGTTTGAAACTCTCACTGG‑3’ |
| 12 (*ere-oe2*) | S3 | 5‘‑GACCGCGATTTAAATCGGTA‑3’ | 5‘‑GCTCTTTGTTTGAAACTCTCACTGG‑3’ |
| *simulans-oe* | 3 | 5‘‑TAGCTTTCCAAGAGTGCAACAATGTA‑3’ | 5‘‑CTGTTTCTGCTTTTGGACTGG‑3’ |
| *sechellia-oe* | 3 | 5‘‑TAGCTTTCCAGGAGTGCTAC‑3’ | 5‘‑CAGTTTCTGCTTTTTGACTG‑3’ |
| *takahashii-oe* | 3, 5 | 5‘‑GTAAACAAATTTTCAGCCGCGC‑3’ | 5‘‑GTTGCTCTAACTGCGATTTTTGC‑3’ |
| *pseudoobscura-oe* | 3 | 5‘‑GAACCTGCCGCTTAGGCGAATG‑3’ | 5‘‑GTGCACTACAAACTGGGGGGTTG‑3’ |

**Table S4.** **Primers used to PCR amplify CREs used in this study**.

All PCR products were cloned into the S3aG EGFP reporter vector described in Williams et al., 2008 using the restriction enzymes AscI and SbfI.
